# Case report: A reciprocal translocation-free and pathogenic *DUOX2* mutation-free embryo selected by complicated preimplantation genetic testing resulted in a healthy live birth

**DOI:** 10.3389/fgene.2023.1066199

**Published:** 2023-02-17

**Authors:** Biwei Shi, Yinghui Ye

**Affiliations:** Department of Reproductive Endocrinology, Women’s Hospital, Zhejiang University School of Medicine, Hangzhou, China

**Keywords:** reciprocal translocation, next-generation sequencing, preimplantation genetic testing, SNP-based linkage analysis, live birth, *DUOX2*

## Abstract

Preimplantation genetic testing (PGT) is an effective approach to improve clinical outcomes and prevent transmission of genetic imbalances by selecting embryos free of disease-causing genes and chromosome abnormalities. In this study, PGT was performed for a challenging case in which a couple simultaneously carried a maternal subchromosomal reciprocal translocation (RecT) revealed by fluorescence *in situ* hybridization involving the chromosome X (ChrX) and heterozygous mutations in dual oxidase 2 (*DUOX2*). Carriers of RecT are at increased risk for infertility, recurrent miscarriages, or having affected children due to the unbalanced gametes produced. *DUOX2* mutation results in congenital hypothyroidism. Pedigree haplotypes for *DUOX2* was constructed after the mutations were verified by Sanger sequencing. Since male carriers of X-autosome translocations may exhibit infertility or other abnormalities, pedigree haplotype for chromosomal translocation was also constructed to identify embryo with RecT. Three blastocysts were obtained by *in vitro* fertilization and underwent trophectoderm biopsy, whole genomic amplification, and next-generation sequencing (NGS). A blastocyst lacking copy number variants and RecT but carrying the paternal gene mutation in *DUOX2*, c.2654G>T (p.R885L) was used for embryo transfer, resulting in a healthy female infant whose genetic properties were confirmed by amniocentesis. Cases containing RecT and single gene disorder are rare. And the situation is more complicated when the subchromosomal RecT involving ChrX cannot be identified with routine karyotype analysis. This case report contributes significantly to the literature and the results have shown that the NGS-based PGT strategy may be broadly useful for complex pedigrees.

## Introduction

Hydrogen peroxide (H_2_O_2_) oxidizes iodide by thyroid peroxidase during thyroid hormonogenesis (De Deken and Miot, 2019). Genetic alterations in the H_2_O_2_-generating system have been implicated in the pathogenesis of congenital hypothyroidism (CH), one of the most frequent congenital endocrine disorders in childhood (Weber et al., 2013). Dual oxidase 2 (DUOX2) is one of the main enzymes in the H_2_O_2_-generating system. DUOX2 defects are one of the leading causes of dyshormonogenesis (Muzza and Fugazzola, 2017). Human *DUOX2* is located on chromosome 15 (15q15.3) and spans 21.5 kb containing 34 exons, of which 33 are coding exons and encode a protein with 1548 amino acid residues (De Deken and Miot, 2019). Mutations in *DUOX2* have highly variable phenotypic effects, ranging from transient to permanent forms of CH (Weber et al., 2013). Reciprocal translocation (RecT) is a category of chromosomal abnormality in which reciprocal exchange occurs between partial arms of any two chromosomes. It is the most common chromosomal rearrangement affecting humans, with an estimated incidence of 0.16%–0.20% of live births (Morin et al., 2017). Most cases with RecT exhibit a normal phenotype, as key genes are not lost (Zhang et al., 2016). However, due to high rates of unbalanced gametes, patients have high risks of infertility as well as chromosomal abnormalities in pregnancy, leading to recurrent spontaneous abortion or affected offspring (Scriven et al., 1998; Fiorentino et al., 2011).

Preimplantation genetic testing (PGT), a branch of *in vitro* fertilization technology, includes testing for monogenic disorders (PGT-M), structural rearrangements (PGT-SR), and aneuploidy (PGT-A) (Zegers-Hochschild et al., 2017). In the present study, we studied the pedigree of a family in which the proband harbored compound heterozygous mutations in *DUOX2* and chromosomal aneuploidies in partial chromosomes X and 18. To simultaneously address the monogenetic disorders and chromosomal abnormalities in the family, a PGT strategy based on whole-genome amplification (WGA), next-generation sequencing (NGS), and a single-nucleotide polymorphism (SNP)-based linkage analysis was applied to select embryos free of pathogenic *DUOX2* mutations, screen chromosomal aneuploidies, and distinguish between translocation carrier embryos and normal embryos. A healthy baby was born at term.

## Material and methods

### Case description

The proband was a male child hospitalized for cleft palate and neonatal septicemia. Clinical examination identified multiple problems: a large heart, CH, developmental delay, extensive neurogenic damage involving the limbs, hearing impairment, and photophobia. Initially, thyroid-stimulating hormone (TSH) levels of the proband was 48.83 mIU/L (27 December 2016). After thyroxine treatment, his TSH levels decreased to 2.22 mIU/L (11 April 2017) (Supplementary Figure S1). But the proband still presents intellectual disabilities, hearing impairment, etc. The proband was the first pregnancy of unrelated healthy parents.

Karyotype analysis of the proband and his parents revealed normal results (Supplementary Figure S2). Whole exome sequencing (WES) and copy number variants (CNV) sequencing were performed simultaneously for the family. The average coverage of WES was more than 99%. WES results revealed that mother (29 years) of the proband was a carrier of a heterozygous mutation in *DUOX2*, c.1588A>T (p.K530X), his father (30 years) carried a heterozygous mutation in *DUOX2*, c.2654G>T (p.R885L), and the proband harbored both heterozygous mutations in *DUOX2*, which is associated with CH. *DUOX2*, c.1588A>T (p.K530X) was classified as pathogenic (PVS + PM + PP), and *DUOX2*, c.2654G>T (p.R885L) was classified as likely pathogenic (2PM+2PP) in accordance with the ACMG guidelines. The gene variant pedigree of the family was constructed (Supplementary Figure S3). The results of CNV sequencing (0.6× sequencing depth) revealed that the proband exhibited an Xq28-q28 duplication (5.59 Mb × 2) and 18q22.2-q23 deletion (9.85 Mb × 1) and the CNVs of the parents were normal (Supplementary Figure S4). According to CNV results, fluorescence *in situ* hybridization (FISH) was performed for the parents and the results showed that the mother was a carrier of a RecT between ChrX and Chr18 [t (X; 18) (q28; q22.2)] (Supplementary Figure S5). With the help of PGT technology, the couple hoped to give birth to a healthy baby (babies) free of pathogenic *DUOX2* mutations and RecT.

### Pedigree haplotype construction for *DUOX2* and chromosomal translocation

Genomic DNAs from the proband and his parents were extracted from blood samples. NGS was performed on the Illumina NextSeq 550 platform (San Diego, CA, United States). The *DUOX2* mutations in the family were verified by Sanger sequencing. Haplotype information for alleles in linkage with wild-type/mutant and translocation/no translocation alleles was established based on informative SNPs.

### Stimulation protocol

The cycles were subjected to controlled ovarian hyperstimulation protocols. Ovarian stimulation was performed using a GnRH antagonist protocol. Transvaginal sonography and serial E2 levels were used to monitor ovarian follicular development. Once a dominant follicle reached 19–20 mm, 10,000 IU of human chorionic gonadotropin (hCG) was administered. Thirty-6 hours later, transvaginal ultrasound-guided oocyte retrieval was performed (Ye et al., 2021).

### Embryo biopsy, cryopreservation, and thawing

Embryo biopsies were performed after intracytoplasmic sperm injection (ICSI) and embryo culture. According to the Gardner scoring system (Gardner et al., 2000), trophectoderm (TE) cell biopsy of high-grade blastocysts (greater than 4CB or 4BC) was performed on day 5 or 6 of the culture. The biopsied TE cells were immediately washed in 1% polyvinylpyrrolidone and transferred into sample collection tubes (Yikon Genomics, Shanghai, China) (Ye et al., 2021). After the biopsy, blastocysts were cryopreserved by vitrification using the Oocyte/Embryo Vitrification Kit VT101 (Kitazato, Tokyo, Japan). According to the PGT results, normal blastocysts were thawed using the Oocyte/Embryo Thaw Kit VT102 (Kitazato). After thawing, the blastocytes were cultured in a G-2 medium (Vitrolife, Gothenburg, Sweden) for 2 h before embryo transfer.

### WGA, specific PCR, and NGS

An improved MALBAC (multiple annealing and looping-based amplification cycles) WGA strategy was used to amplify the genomes of biopsied TE cells. The specific steps of WGA with MALBAC are described previously (Ye et al., 2021). Two mutation sites in *DUOX2*, c.1588 A>T (p.K530X) and c. 2654 G>T (p.R885L) were targeted from the WGA product. The specific polymerase chain reaction (PCR) products were mixed with the corresponding WGA product to construct a library using the NGS Library Preparation Kit (Yikon Genomics). The mixture was sequenced by NGS with a 2 × sequencing depth. The sequencing data was used to separate the mutant and wild-type haplotypes, to detect CNVs and to identify the translocation-carrying allele.

### SNP-based linkage analysis

For *DUOX2* mutations, using the pedigree haplotyping results as a reference, the SNP readouts at the positions adjacent to the mutant loci allowed the identification of the mutation-carrying allele in embryos. Mutations were also validated by Sanger sequencing. After the translocation-carrying allele was identified in the proband, the same SNP markers were used to corroborate whether the embryo carried chromosomally balanced RecT. The bioinformatics analysis was performed using ChromGo (Yikon Genomics).

### Confirmation of pregnancy and prenatal diagnosis

Pregnancy was confirmed by the level of serum β-hCG 14 days after frozen-thawed embryo transfer (FET) and by the presence of a gestational sac on ultrasound 35 days after FET. Amniotic fluid was collected for karyotyping, chromosomal aneuploidy detection, and *DUOX2* mutation analysis. G-band karyotyping, an SNP array analysis, and Sanger sequencing were performed for the above three examinations.

## Results

**FIGURE 1:**
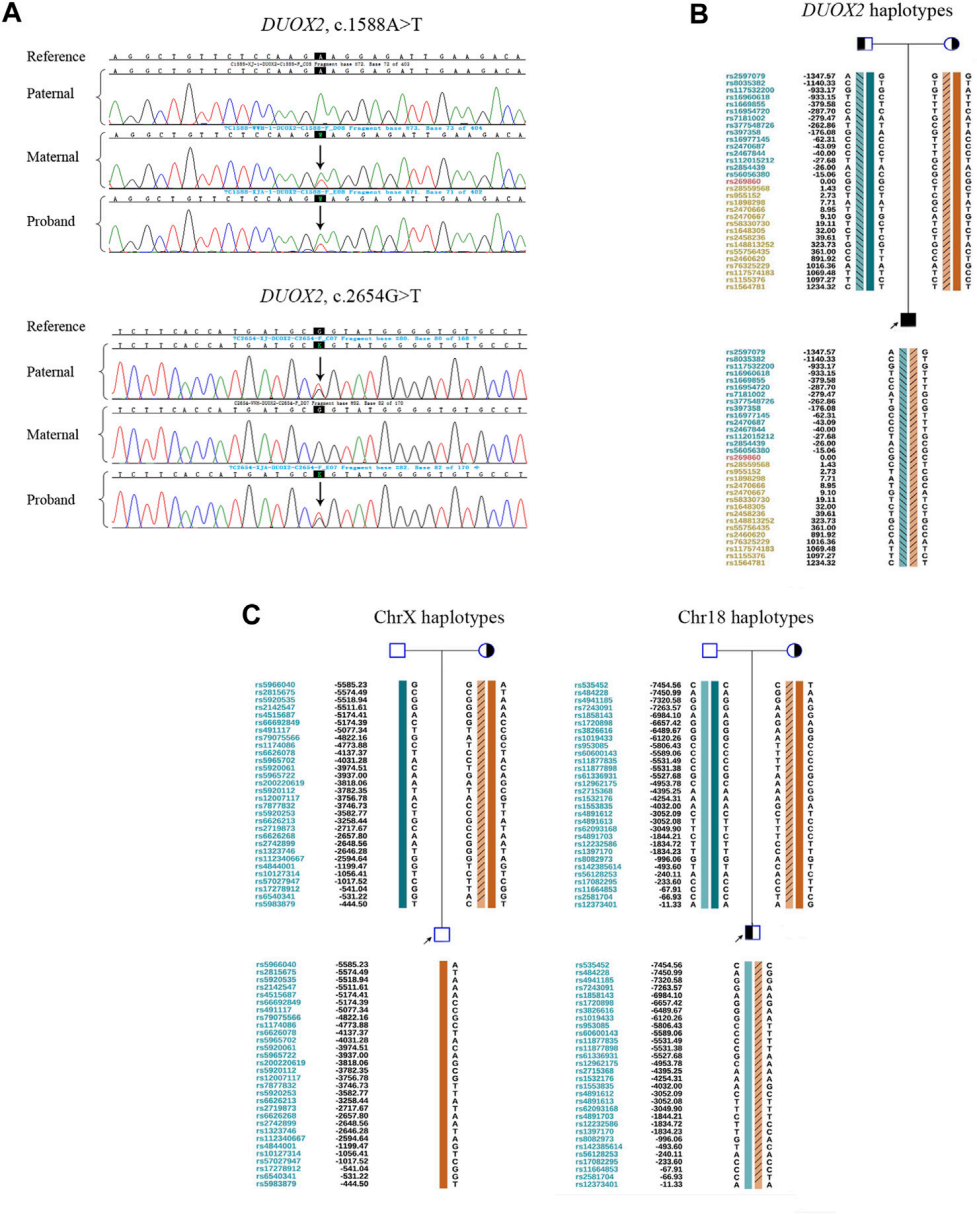
Preliminary experiments. **(A)** Sanger sequencing results for validation of the *DUOX2* mutations in c.1588A>T (p.K530X) and c.2654G>T (p.R885L) in family members. The black background indicates the targeted mutation sites. Arrows denote mutations. **(B)** Pedigree haplotyping analysis based on informative SNPs flanking *DUOX2*. The arrow indicates the proband. Circles and squares indicate females and males, respectively. The filled symbol represents the affected patient. Half-filled symbols represent *DUOX2* mutation carriers. Haplotypes are represented by colorful bars combined or not combined with diagonal lines. The dark green bar indicates a paternal wild-type haplotype. The light green bar combined with diagonal lines denotes a paternal mutation: *DUOX2*, c.2654G>T (p.R885L). The dark orange bar denotes a maternal wild-type haplotype. The light orange bar combined with diagonal lines indicates a maternal mutation: *DUOX2*, c.1588A>T (p.K530X). **(C)** Pedigree haplotypes around chromosomal breakpoint regions. The arrow indicates the proband. Circles and squares indicate females and males, respectively. Half-filled symbols represent derivative chromosome carriers. In ChrX, the dark green bar indicates the normal paternal ChrX haplotype. The dark orange bar denotes the normal maternal ChrX haplotype. Light orange bars with diagonal lines represent the derivative ChrX haplotype. In Chr18, the dark and light green bars indicate a pair of paternal normal Chr18 haplotypes in two homologous chromosomes. The dark orange bar represents the normal maternal Chr18 haplotype. The light orange bar combined with diagonal lines denotes the derivative Chr18 haplotype.

*DUOX2* variants in the family were validated by Sanger sequencing. The proband was a carrier of compound heterozygous mutations c.1588 A>T (p.K530X) and c. 2654 G>T (p.R885L). His mother carried the heterozygous mutation c.1588 A>T (p.K530X), and his father carried the heterozygous mutation c. 2654 G>T (p.R885L) (Figure 1A). In total, 187 SNPs within 2 Mb of the mutation site were evaluated by NGS. Pedigree haplotypes with linkage to the wild-type allele and mutation alleles c. 2654 G>T (p.R885L) or c.1588 A>T (p.K530X) were separated (Figure 1B). Translocation breakpoints in the proband were identified based on the copy number. The positions of breakpoints were ChrX: 149,327,063 and Chr18: 68,144,137. Subsequently, 60 SNP markers flanking the chromosomal breakpoints (within 1 Mb) were used to identify the translocation-carrying alleles in the proband and his mother (Figure 1C).

**FIGURE 2:**
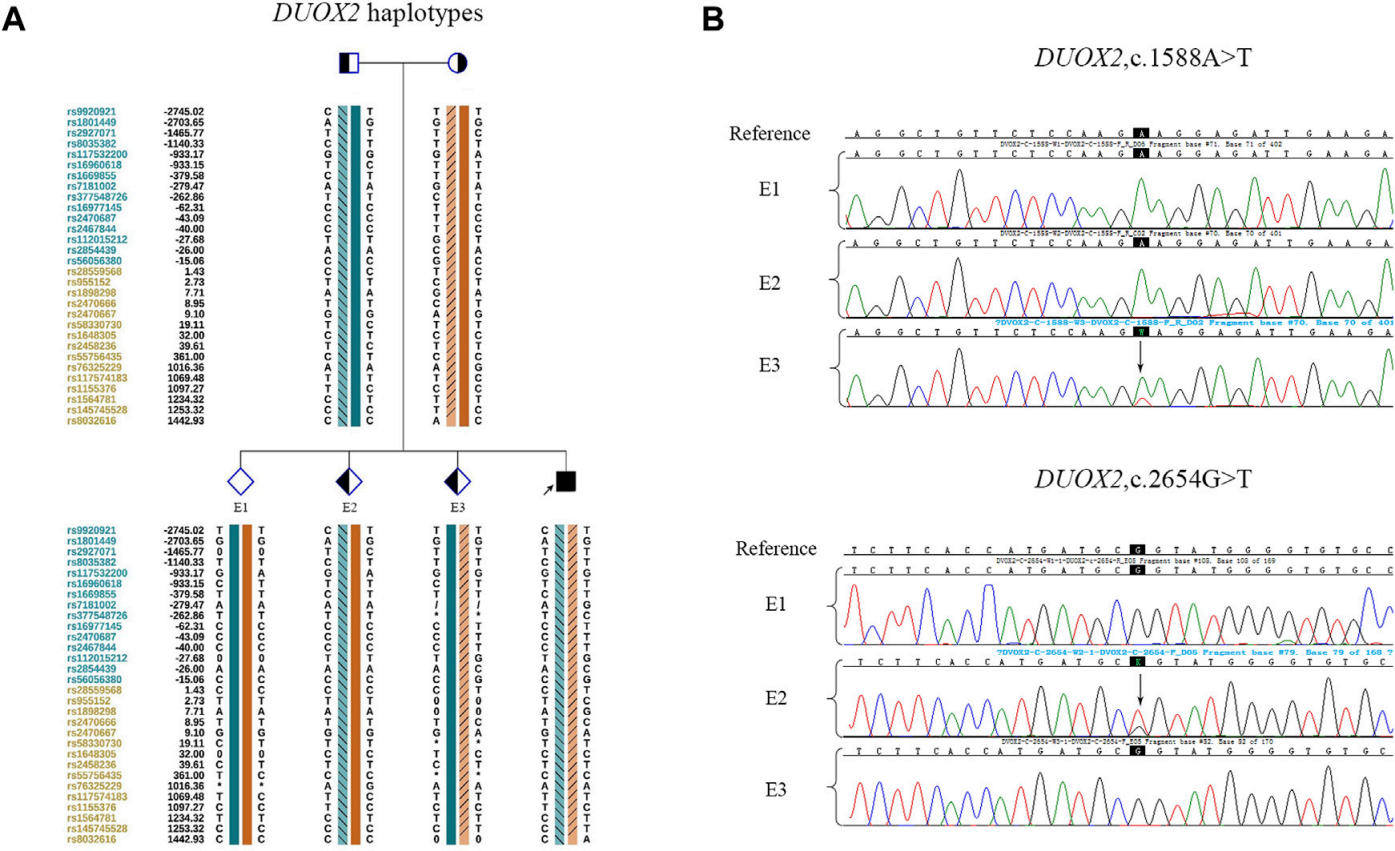
Linkage analysis and mutation detection in embryos. **(A)** Haplotypes around *DUOX2* of the family members and embryos. Diamonds indicate embryos, and the interpretation of other symbols and bars is explained in the legend of Figure 1B. E1 inherited both wild-type haplotypes from parents. E2 inherited the paternal mutation and maternal wild-type haplotype. E3 inherited the paternal wild-type haplotype and maternal mutation. **(B)** The mutations in embryos were identified by Sanger sequencing. The results were consistent with those of the SNP-based linkage analysis.

**FIGURE 3:**
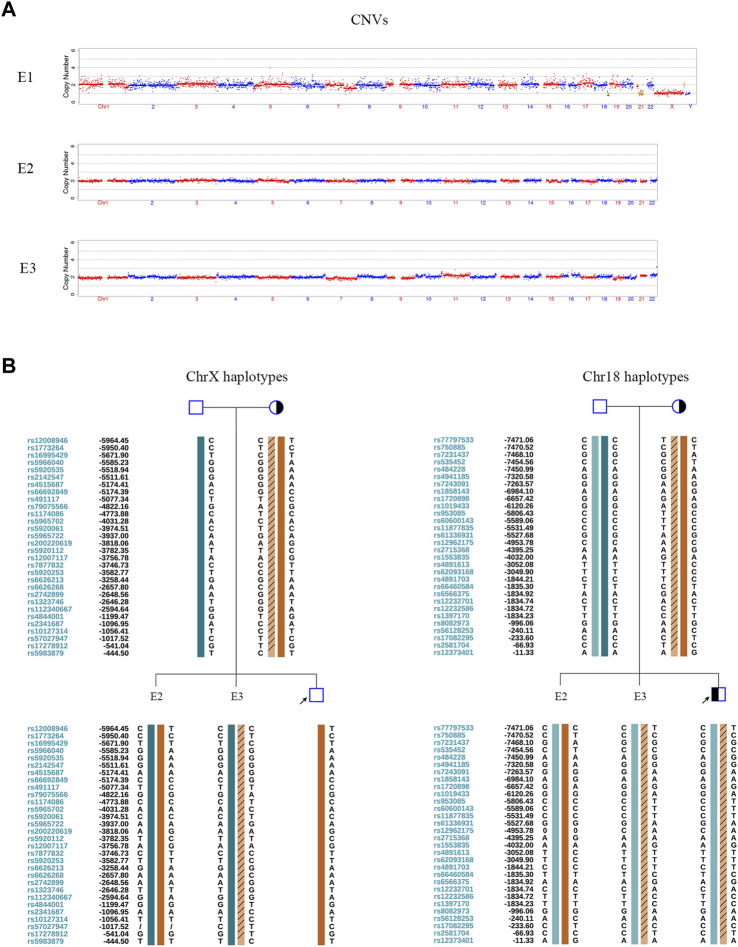
CNV tests and SNP-based linkage analysis for RecT detection in embryos. **(A)** Embryonic CNV results. E1 was unbalanced. E2 and E3 were chromosomally balanced. **(B)** Pedigree haplotyping around chromosomal breakpoints. The interpretation of symbols and bars is explained in the legend of Figure 1C. E2 inherited its two wild-type X chromosomes and two wild-type 18 chromosomes from their parents. It was female and free of RecT. E3 inherited a derivative ChrX and derivative Chr18 from the mother. It carried RecT between ChrX and Chr18.

In the PGT cycle, 12 oocytes were retrieved. Seven oocytes were mature, and all were fertilized. Three fertilized eggs developed into blastocysts with Gardner scores of 3BB, 4BB, and 4BB. The haplotyping results revealed that E1 carried paternal and maternal wild-type haplotypes and was free of *DUOX2* mutations c. 2654 G>T (p.R885L) and c.1588 A>T (p.K530X). E2 carried the paternal *DUOX2* mutation c. 2654 G>T (p.R885L), and E3 was identified as a carrier of the maternal *DUOX2* gene variant c.1588 A>T (p.K530X) (Figures 2A,B). A CNV analysis of these three embryos showed that E1 was chromosomally unbalanced, whereas E2 and E3 were balanced (Figure 3A). An analysis of targeted SNP sites flanking the breakpoint confirmed that E2 inherited wild-type Chr18 and ChrX haplotypes from parents and was free of RecT. E3 inherited translocation-carrying haplotypes of Chr18 and ChrX from the mother. Accordingly, E3 was a carrier of RecT between Chr18 and ChrX (Figure 3B). Based on these findings (i.e., E1 lacked mutations but was chromosomally unbalanced, E2 lacked RecT but was a carrier of the paternal gene mutation, and E3 inherited RecT and the heterozygous mutation from the mother), E2 was chosen for embryo transfer.

**FIGURE 4:**
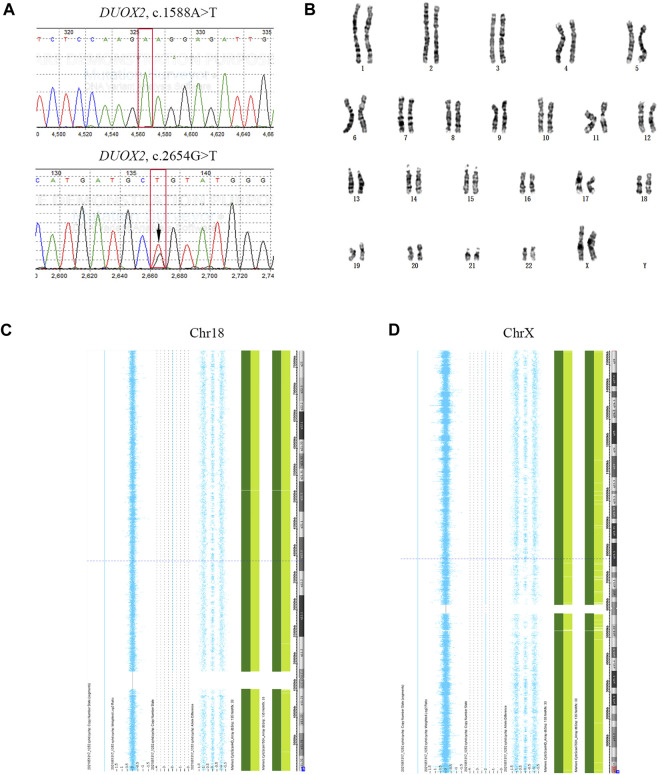
Prenatal diagnostic results. **(A)** Sanger sequencing results for fetal *DUOX2* mutations. Red boxes represent targeted mutation sites. The arrow indicates the *DUOX2*, c.2654G>T (p.R885L) mutation. **(B)** Amniotic fluid karyotyping of the fetus, revealing a normal karyotype. **(C, D)** Results of SNP array-based comprehensive chromosome screening.

The serum β-hCG level of the mother was 1607 IU/L 14 days after FET. A gestational sac was detected by ultrasound 35 days after FET. Amniocentesis was performed at gestational week 20 to confirm the genetic properties of the fetus. The results were consistent with the PGT results for E2 (Figure 4). A healthy female infant weighing 3.25 kg was delivered by caesarean section at gestational week 38.5.

## Discussion

We diagnosed a complex, rare case in which a family simultaneously exhibited pathogenic compound heterozygous gene mutations and a chromosomal abnormality involving ChrXq. We utilized PGT based on NGS and an SNP linkage analysis to exclude monogenic disorders and RecT. Two sets of informative SNPs obtained by NGS were utilized to detect gene mutations and RecT in embryos. The selection of a RecT-free but monoallelic *DUOX2* mutation-carrying embryo resulted in a healthy live birth.

Although the Xq28-q28 duplication and 18q22.2-q23 deletion were detected in the proband by a CNV sequencing, the parents presented normal G-band karyotyping. A subchromosomal RecT (<10 Mb) in the mother was confirmed by FISH. G-band karyotyping is a routine laboratory test used for genetic diagnosis with a resolution of 5–10 Mb. However, given the variation in banding resolution among prenatal preparations, 10–20 Mb or greater is a more realistic threshold for detection in conventional karyotype analyses (Levy and Wapner, 2018; Kamath et al., 2022). CNV analyses offer additional diagnostic benefits by revealing sub-microscopic imbalances or CNVs that are substantially small to be detected on a standard G-banded chromosome preparation (Levy and Wapner, 2018). Several studies have demonstrated that, for the cases with normal parental karyotypes, the inspection of products of conception by CNV analyses is required to identify sub-microscopic chromosomal abnormalities in parents (Qian et al., 2018; Chen et al., 2021).

RecT carriers usually present a normal phenotype; however, they have elevated risks of reproductive abnormalities, including infertility, spontaneous abortion, and congenital disabilities. The PGT strategy based on NGS and an SNP linkage analysis to exclude RecT is expected to be an effective approach. In this case, RecT-carrying male offspring are at risk of disorders related to X-autosome translocation. X-autosomal translocations are rare and generally of the maternal origin or arise *de novo* (Kalz-Fuller et al., 1999). Both female and male carriers of X-autosomal translocations may present disorders. In female carriers, normal X chromosome inactivation leads to multiple anomalies and/or intellectual disabilities due to the derivative X chromosome in the active state (Ma et al., 2003). Breakpoints in the Xq13-q26 region are associated with infertility, ranging from gonadal dysgenesis to premature ovarian failure (Madan, 1983; Choi et al., 2020). Male carriers of X-autosome translocations may exhibit a disturbance in spermatogenesis, leading to severe subfertility or infertility (Madan, 1983). This is explained by the fact that the derivative X chromosome may interfere with sex vesicle formation, leading to meiotic disturbance and, consequently, to spermatogenetic arrest (Perrin et al., 2008). Although the RecT-carrying female in our case had a normal phenotype, the risk of disorders related to an X-autosome translocation in male offspring cannot be ruled out.

A 2002 study demonstrated that biallelic inactivating mutations in *DUOX2* resulted in the complete disruption of thyroid hormone synthesis and were associated with severe and permanent CH. Monoallelic mutations are associated with mild, transient hypothyroidism caused by insufficient thyroidal hydrogen peroxide production (Moreno et al., 2002). Several later studies have provided evidence that the transient or persistent nature of the hypothyroid phenotype is not directly related to the number of inactivated *DUOX2* alleles (Maruo et al., 2008; Ohye et al., 2008; Hoste et al., 2010). In 2018, a study in China revealed that *DUOX2* is the causative gene in patients with biallelic *DUOX2* mutations (containing compound heterozygous and homozygous mutations); however, *DUOX2* may not be the causative gene for patients carrying a monoallelic *DUOX2* mutation (Sun et al., 2018). Referring to the relevant literature and considering the normal phenotypes of the parents who carried monoallelic *DUOX2* mutations, we decided to transfer E2.

Genome-wide technologies have replaced FISH and PCR over the last decade (ESHRE PGT-SR/PGT-A Working Group et al., 2020). Genome-wide testing is typically applied to TE biopsy and used with a freeze-all strategy. TE biopsy can be applied to multiple cells and enables subsequent improvements in the accuracy of results with decreased amplification errors (Veiga et al., 1997; Sullivan-Pyke and Dokras, 2018). NGS-based SNP haplotyping avoids pitfalls associated with allele dropout and improves the accuracy of PGT-M (Chen et al., 2019). As the cost of NGS continues to decline, PGT is moving towards a sequencing-based all-in-one solution for PGT-M, PGT-SR, and PGT-A (De Rycke and Berckmoes, 2020; Wang et al., 2020), which would significantly simplify the procedure of PGT and broaden the applicable situations of the PGT technique.

In conclusion, we applied NGS and linkage analysis for PGT in a case of a complex pedigree. We successfully selected an appropriate embryo that resulted in a healthy live birth, and the clinical results proved the effectiveness of our diagnostic strategy.

## Data Availability

The original contributions presented in the study are publicly available. This data can be found here: https://gosspublic.alicdn.com/oss-browser/1.16.0/oss-browser-win32-x64.zip?spm=a2c4g.11186623.0.0.43179c1d6w6l8u&file=oss-browser-win32-x64.zip.
