# Supplementary material for: Case report: A reciprocal translocation-free and pathogenic DUOX2 mutation-free embryo selected by complicated preimplantation genetic testing resulted in a healthy live birth
Source: Front Genet. 2023 Feb 17;14:1066199. doi: 10.3389/fgene.2023.1066199 (PMC9982009; doi:10.3389/fgene.2023.1066199)
Supplement: Supplementary file 1 [file Table1.DOCX]

Supplementary Material

**Case Report:** **A reciprocal translocation-free and pathogenic *DUOX2* mutation-free embryo selected by complicated preimplantation genetic testing resulted in a healthy live birth**

**Biwei Shi, Yinghui Ye****^*^**

*** Correspondence:** Yinghui Ye: [yeyh1999@zju.edu.cn](mailto:yeyh1999@zju.edu.cn)

**Supplementary Figures**

**Supplementary Figure S1.** Thyroid function tests of the proband. **(A)** Thyroid-stimulating hormone (TSH) levels of the proband was 48.83 mIU/L (Dec 27, 2016). **(B)** After thyroxine treatment, his TSH levels decreased to 2.22 mIU/L (April 11, 2017).

**Supplementary Figure S2.** Karyotype figures of the family members. **(A)** Karyotype figure of the proband: 46, XY. **(B)** Maternal karyotype figure: 46, XX. **(C)** Paternal karyotype figure: 46, XY.

**Supplementary Figure S3.** Pedigree of the family. The arrow indicates the proband. Circles and squares indicate females and males, respectively. The filled symbol represents the affected patient. Half-filled symbols represent *DUOX2* mutation carriers.

**Supplementary Figure S4.** CNV sequencing results of the family. The results of CNV sequencing (0.6× sequencing depth) reveal that the proband exhibit an Xq28-q28 duplication (5.59 Mb×2) and 18q22.2-q23 deletion (9.85 Mb×1) and the CNVs of the parents are normal.

**Supplementary Figure S5.** Fluorescence in situ hybridization (FISH) results of parents. **(A)** and **(B)** FISH tests for mother reveal that she is a carrier of a RecT between ChrX and Chr18 [t(X; 18)(q28; q22.2)]. **(A)** Three probes are used in the test: CEPX Aqua, Xp SG and Xq SO. **(B)** Three types of probes used are: CEP18 Aqua, 18p SG and 18q SO. **(C)** and **(D)** FISH results of father reveal that he is free of RecT. **(C)** Three probes are used in the analysis: CEP18 Aqua, 18p SG and 18q SO. **(D)** The test is performed by probes CEPX Aqua, Xp SG and Xq SO. All types of probes used in analyses are Vysis FISH probes (Abbott, Abbott Park, Illinois, U.S.A.).
