# Supplementary figures and images for: Case report: A reciprocal translocation-free and pathogenic DUOX2 mutation-free embryo selected by complicated preimplantation genetic testing resulted in a healthy live birth
Source: Front Genet. 2023 Feb 17;14:1066199. doi: 10.3389/fgene.2023.1066199 (PMC9982009; doi:10.3389/fgene.2023.1066199)

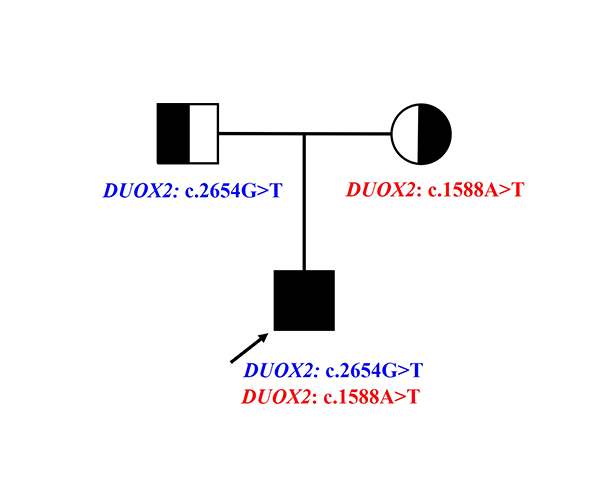

Supplement: Supplementary file 2 [file Image3.TIF]

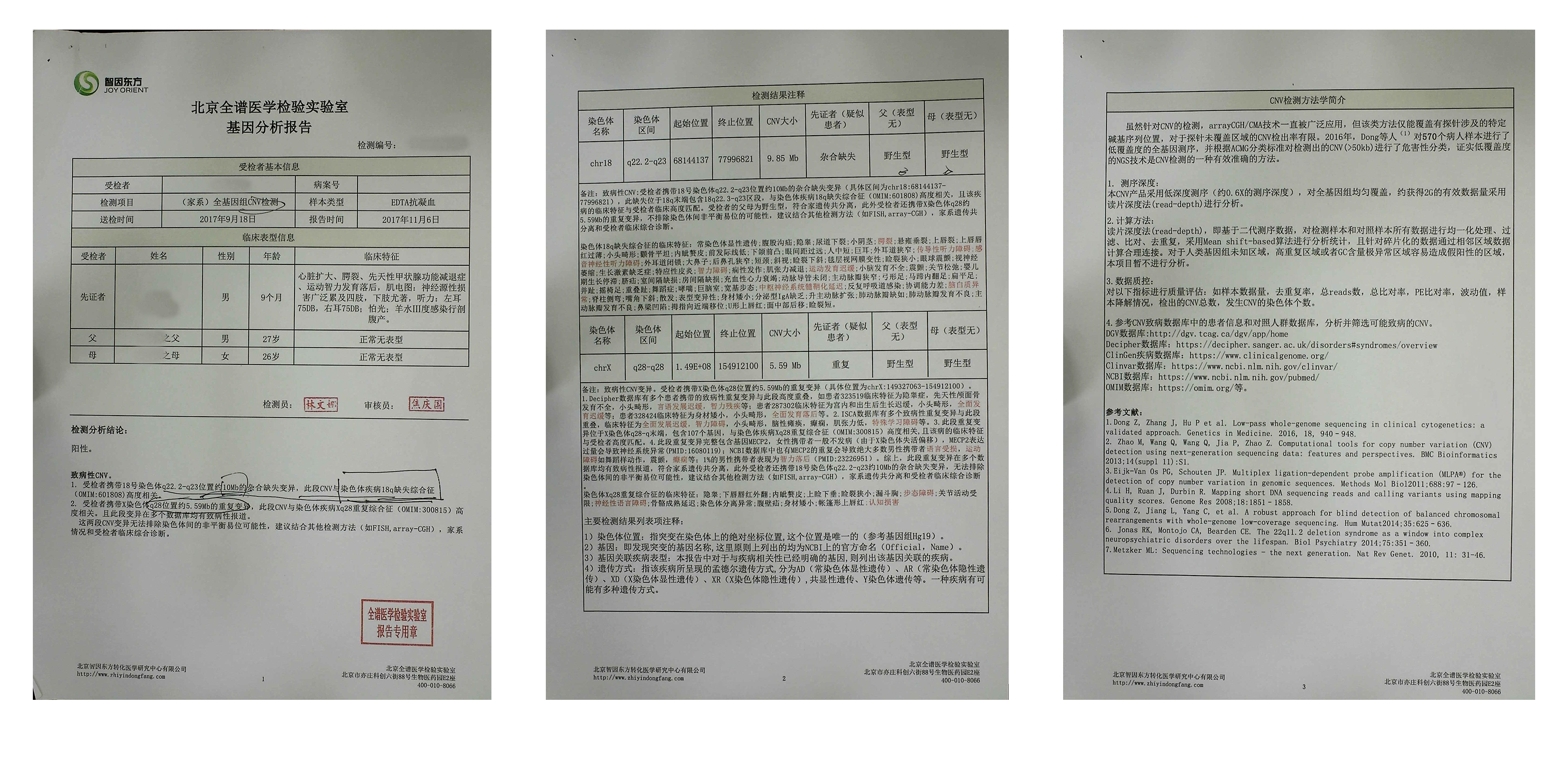

Supplement: Supplementary file 3 [file Image4.TIF]

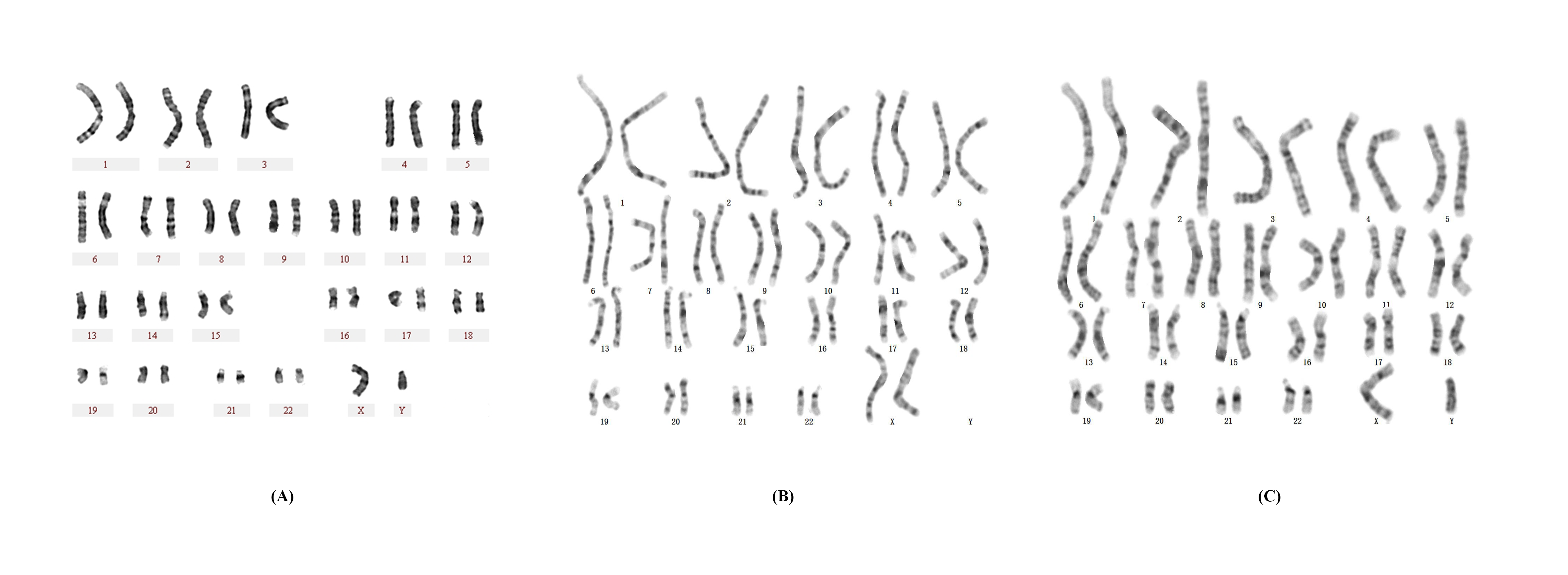

Supplement: Supplementary file 4 [file Image2.TIF]

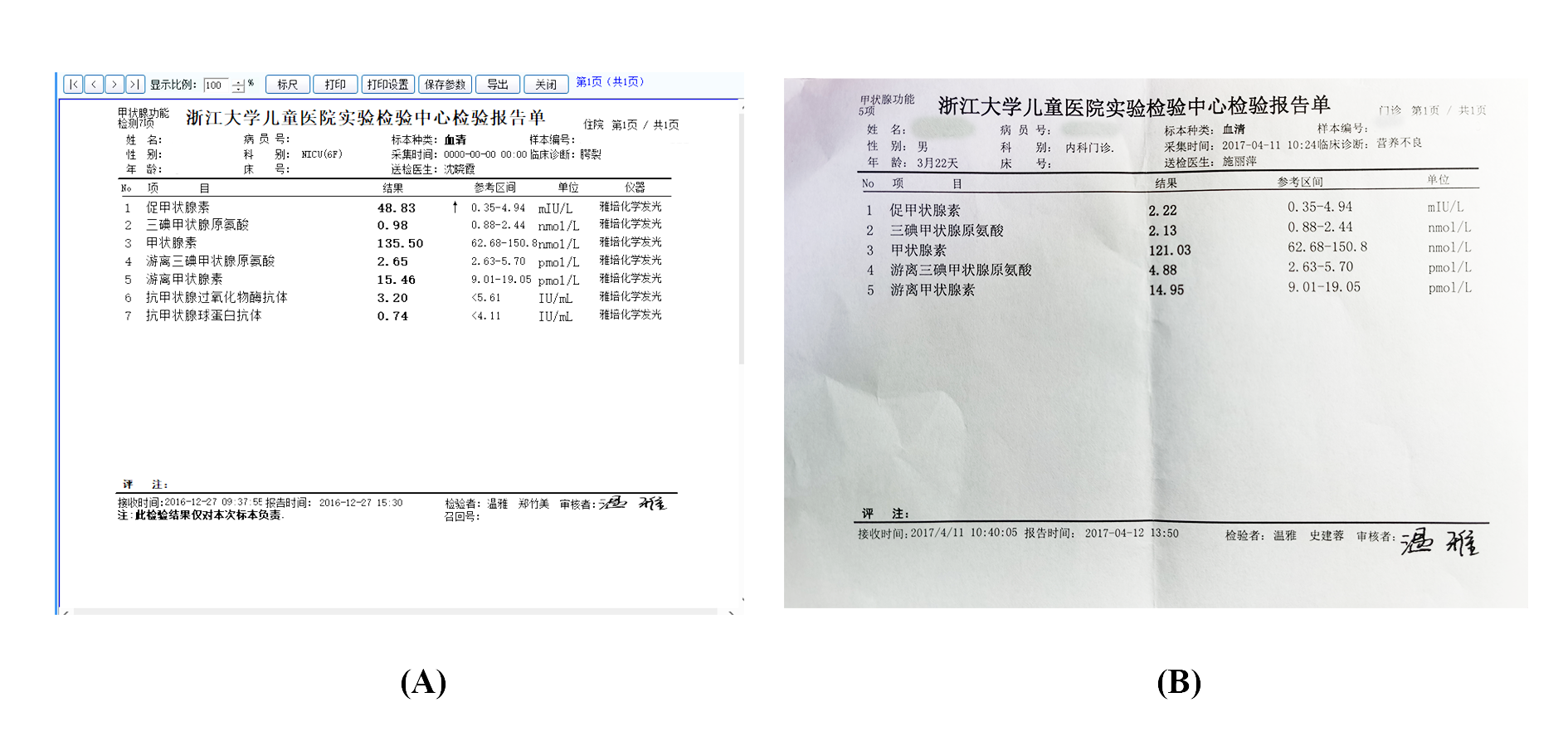

Supplement: Supplementary file 5 [file Image1.TIF]

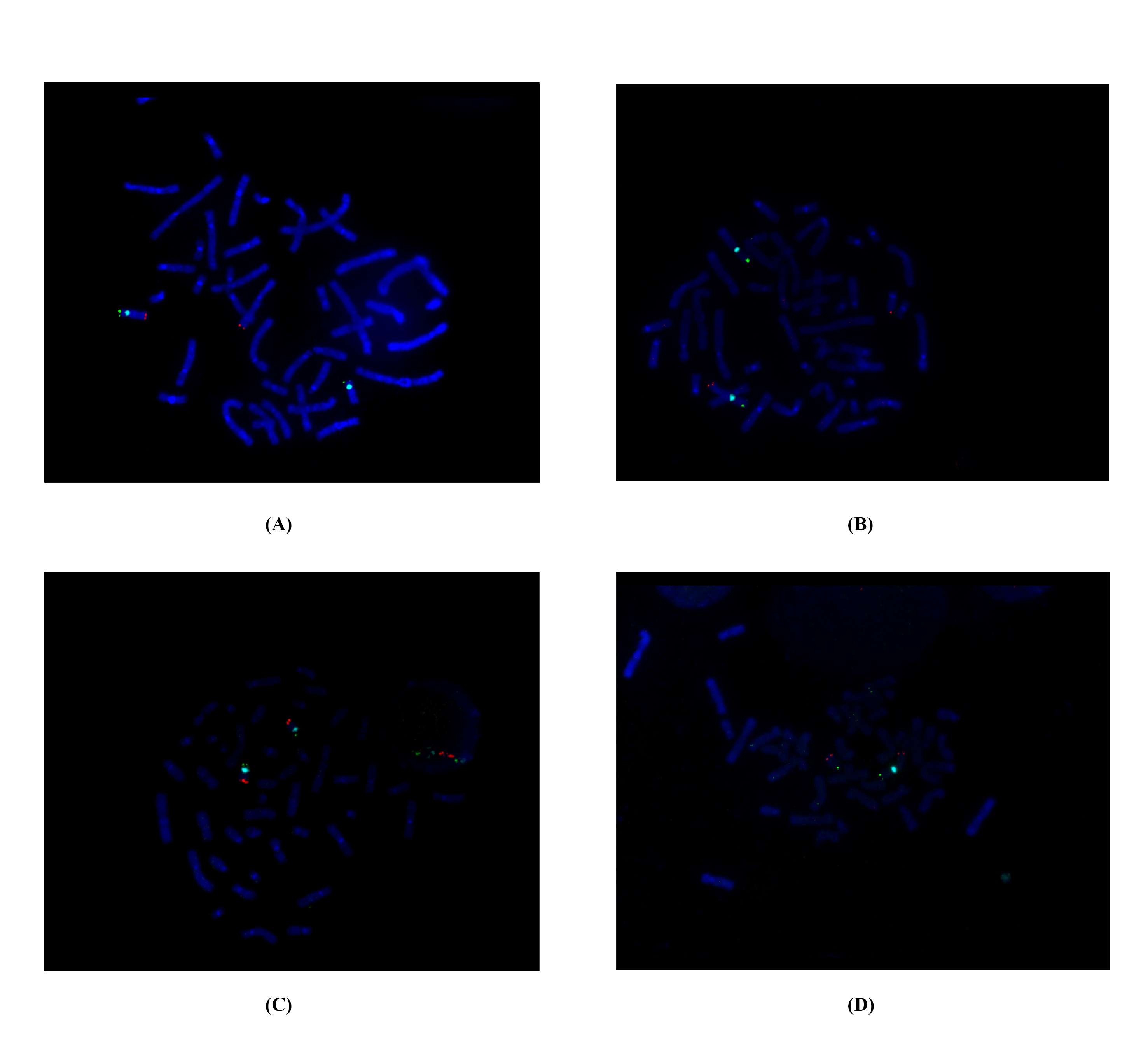

Supplement: Supplementary file 6 [file Image5.TIF]
